# Promoting vaccination in the province of Québec: the PromoVaQ randomized controlled trial protocol

**DOI:** 10.1186/s12889-019-6468-z

**Published:** 2019-02-06

**Authors:** Arnaud Gagneur, Caroline Quach, François D. Boucher, Bruce Tapiero, Philippe De Wals, Anne Farrands, Thomas Lemaitre, Nicole Boulianne, Chantal Sauvageau, Manale Ouakki, Virginie Gosselin, Dominique Gagnon, Geneviève Petit, Marie-Claude Jacques, Ève Dubé

**Affiliations:** 1 Centre de recherche du CHUS, Sherbrooke, QC Canada; 2 Pediatrics Department, Neonatology Unit, Centre hospitalier universitaire de Sherbrooke, Université de Sherbrooke, 3001, 12e Avenue Nord, Sherbrooke, QC J1H 5N4 Canada; 3 CHU Sainte Justine, Université de Montréal, Montréal, QC Canada; 4 McGill University Health Centre Research Institute – Vaccine Study Centre, Montréal, QC Canada; 5 Centre de recherche du Centre Hospitalier Universitaire de Québec, Québec, Canada; 6 Department of Social and Preventive Medicine, Laval University, Québec, Canada; 7 Institut national de santé publique du Québec, Québec, Canada; 8 Direction de santé publique du CIUSSSE – CHUS, Département des sciences de la santé communautaire, Université de Sherbrooke, Sherbrooke, QC Canada; 9 Institut universitaire de première ligne en santé et services sociaux du CIUSSSE – CHUS, Sherbrooke, QC Canada

**Keywords:** Motivational interviewing, Vaccination coverage, RCT, Province of Québec

## Abstract

**Background:**

Vaccination has a huge public health impact. Maintaining vaccine coverage is key to avoid the devastating consequences of resurgence. In the Province of Québec, vaccine coverage in young children are sub-optimal, mostly due to ambivalence toward vaccine safety and efficacy. We previously conducted a regional study in the Québec’s Eastern Townships region, the PromoVac Study, to test a new educational intervention, based on motivational interviewing techniques, aimed at promoting infant vaccination. This first study evidenced that the intervention led to a marked increase in mothers’ intention to vaccinate, and vaccine coverage in their infants. The current study protocol aims at scaling up these results at a provincial level using a randomized controlled trial design.

**Methods:**

This pragmatic, randomized, controlled, parallel-group clinical trial will compare the effectiveness of the motivational interviewing to an educational intervention, including the distribution of an information flyer as standard of care on vaccination coverage in four maternity wards across the Province of Québec (PromovaQ). Adult mothers of children born in participating maternity wards were recruited between March 2014 and February 2015. Vaccination coverage will be assessed at 3-years of age, thus the trial is expected to be completed in March 2019. Statistical analyses will be conducted under the intention-to-treat principle. Vaccine coverage will be analyzed using Chi-squared distribution testing and logistic regression to identify determinant factors. Secondary outcomes will include vaccine hesitation and intention scores, mother’s knowledge, attitudes and beliefs about immunization, and psychosocial determinants of intention to vaccinate.

**Discussion:**

In the case results of this Provincial RCT be confirmed, serious consideration should then be given by Ministry of Health authorities to the possible implementation of MI-based strategies across provincial maternity wards. To ensure adequate input and secure implementation, study design and results will be reviewed with relevant stakeholders, including the children’s families, and provincial and regional decision-makers. Results will be adapted and shared with all stakeholders.

**Trial registration:**

ClinicalTrials.gov NCT02666872 (Retrospectively registered as January 28, 2016).

## Background

Vaccination has successfully curbed the mortality and morbidity of numerous vaccine-preventable diseases [1]. To keep the incidence of such diseases in check, high levels of vaccine coverage must be maintained, including among children who are most vulnerable. Although vaccine coverage in young children is high in the Province of Québec (Canada), levels are sub-optimal relative to provincial public health program targets. For instance, in 2014, complete vaccination coverage at age 2 years was 71% (85% if excluding rotavirus vaccine), compared to the 95% target [2]. Although the prevalence of most vaccine-preventable diseases remains currently low in the Province of Québec, the transmission of certain vaccine-preventable diseases proceeds uninterrupted. A decline in vaccine coverage could therefore lead to resurgence of currently controlled vaccine-preventable diseases. As an example, a measles outbreak that occurred in the Province of Québec in 2011 resulted in identification of 750 cases, mainly among non-fully immunized children and adolescents [3].

In addition, beyond sub-optimal vaccine coverage, another important issue is off-schedule immunization [4–7]. Delay in the 2-month vaccines was significantly associated with incomplete vaccine coverage at 15 and 24 months [4–7]. One consensus reached by Canadian experts is that late vaccination is defined by a one-month delay past the recommended schedule [8]. In the Province of Québec, late-vaccination management indicators were introduced in 2006 to collect data on late immunization at one week, two weeks, and one month passed the scheduled date for vaccination [9]. Previous surveys of vaccine coverage for 1- and 2-year-old children showed that only 17 to 36% of children aged 24 months had received all of their vaccines within one month of the recommended age [4, 10, 11].

Public health targets for vaccine coverage remain elusive and the persistence of late vaccination are both explained, in parts, by a lack of confidence on the part of the public. As it turns out, vaccination is currently a victim of its own past success. Indeed, the fear associated to vaccine safety has risen steadily among the population as its level of exposure to vaccine-preventable diseases has fallen [12, 13] . Numerous studies report that nearly one third of parents are currently hesitant toward vaccination, [14–19] an often strongly held viewpoint fueled by recent history controversies and media coverage [20].

Vaccine hesitancy and acceptance are intertwined concepts. The World Health Organization defines vaccine hesitancy as the “delay in acceptance or refusal of vaccines, despite availability of vaccine services” [21, 22]. A variety of educational methods, mostly based on providing parents with factual information, have been deployed to address vaccine hesitancy. However, none has as of yet proven effectiveness [23]. A recent Cochrane review of qualitative evidence, assessing parents’ views and experiences of communication about routine childhood vaccination, showed that parents required more information than what they were actually receiving and that simple, context-specific facts should be provided in a timely-manner by a trusty health worker [24]. The take-home message is that while parents want more information, traditional educational methods currently fail to meet their needs according to the literature. This begs the following question: how do we overcome the challenge of providing adapted factual information on vaccination to parents?

One possible approach, originally developed in the context of substance abuse, is the motivational interview (MI). MI has been used to elicit health-related behavioral changes in nutrition, physical activity and smoking [25–28]. MI is basically a patient-oriented communication strategy, used to elicit personal, internal motivation to attitude changes by exploring and solving inherent ambivalence [29]. Our group successfully adapted this approach to develop the PromoVac vaccination promotion program. PromoVac is offered to mothers directly at the maternity wards and is closely tailored to mother’ knowledge and beliefs. The PromoVac regional cohort observational study enrolled 1128 families at the CIUSSSE-CHUS (Centre intégré Universitaire de Santé et de Services Sociaux de l’EStrie – Centre Hospitalier Universitaire de Sherbrooke) maternity ward. Results demonstrated the feasibility and efficacy of the PromoVac MI-based educational intervention to promote both vaccination intention among mothers and vaccine coverage among infants [30, 31]. Results obtained from the PromoVac regional study support a larger, multicenter, randomized controlled trial taking into account the diversity of the Province of Québec’s population.

### Hypothesis

Our working hypothesis is that a vaccination promotion program based on a standardized, MI-based information session held directly at the maternity ward with mothers will increase vaccine coverage of infants in the Province of Québec.

### Objectives

The primary objective is to compare infants’ vaccine coverage at 7 months of age whose mothers, upon recruitment at the maternity ward, were randomized to receive the vaccination promotion study intervention or the standard educational leaflet. The secondary objectives are to compare: 1) infants’ vaccine coverage status (complete, incomplete or not vaccinated) at 3, 5, 13, 19, 24 and 36 months of age; 2) infants’ median age at vaccination for vaccines recommended at 2, 4, 6, 12 and 24 months; 3) mothers’ pre- and post-intervention intention to vaccinate; 4) mothers’ pre- and post-intervention vaccination hesitancy score; 5) maternal’ knowledge, attitudes, beliefs and intention to vaccinate. We also aim to identify intervention implementation obstacles, barriers and facilitators in maternity wards.

## Methods/design

### Design

This pragmatic, unblinded, parallel, randomized, controlled study will compare the impact of a motivational interview (MI) vs the standard of care information leaflet provided to mother of infants born in the past 48 h. The trial recruitment period covered March 2014 to February 2015, with a final outcome measured in 3-year old children. Taking into account the time required to complete data collection and extraction from the appropriate national vaccination and health registries, this trial will be completed in March 2019. Randomization was conducted at the participant/maternal level, using a block size strategy (eight participants/block) to avoid bias. Randomization was stratified by site using a 1:1 allocation ratio to ensure proportionate allocation among sites. Random allocation was investigator-blinded using a web-based system (Dacima Software).

### Study setting

This trial was be conducted in 4 maternity wards in three cities across the Province of Québec, each located in distinct geographical regions: Sherbrooke in the Eastern Townships: the CIUSSSE-CHUS; Montreal: CHU (Centre Hospitalier Universitaire) Ste-Justine and the McGill University Health Centre; Québec city: the CHU de Québec/CHUL. These four sites were selected based on the fact that they collectively serve over 20% of the province’s population and are representative of its ethnic diversity.

### Eligibility

Mothers were deemed eligible to participate if their child was born in one of the participating maternity wards. In the case partners were present at the maternity ward, they were also invited to receive the intervention and be involved with the mothers in assessing the study tools. For the Sherbrooke site, inclusion was limited to mothers living in the Eastern Townships region. For the Québec City site, inclusion was limited to mothers living in the Capitale-Nationale region. Mothers were excluded if < 18 years, if they do not speak either French or English, or should the child presented an unstable condition requiring intensive care management, or should interview be in any way incompatible with the mother’s health.

### Patient involvement

This trial was designed taking into account results from satisfaction survey completed by mothers enrolled in the PromoVac study [32].

### Enrolment

The maternity ward medical staff made initial contact with and ascertained potential study participants during daytime (8 AM to 5 PM) on week days (Monday to Friday). This was done on the day following childbirth, except when that day fell on a Saturday or Sunday. Only when the prospective study participant agreed to meet with the study research assistant, were the project participation details presented. Written informed consent was obtained from each participant prior to starting study participation. To protect identity and privacy, participants were identified by a code number and the code key linking participant’s identity and research file were kept safely by the study investigator during 10 years. Randomization was secured by the research assistant.

### Intervention

Because infant vaccination starts at 2 months of age, it seems appropriate to promote vaccination to mothers very early during their newborn life. Accordingly, maternity wards seemed to offer a convenient setting, as 98% of all Québec infants are born there [33]. Importantly, our feasibility study confirmed that over 85% of families consider the maternity ward an appropriate setting to discuss infant vaccination. Indeed, 97% of study participants confirmed that they would recommend this strategy to other families [32]. Therefore, we established the maternity ward as a key location to dispense the MI-based intervention.

#### Intervention arm

**Fig. 1:**
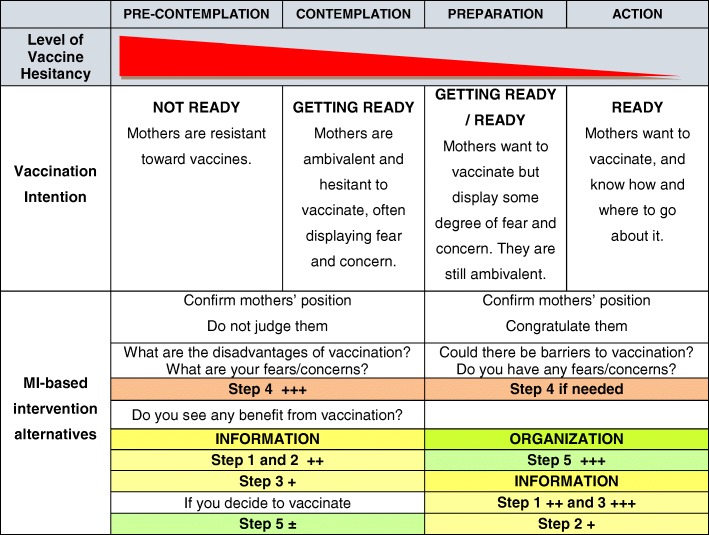
Prochaska’s Stages of Change

The proposed study intervention integrates concepts of the MI and Prochaska’s stages of change [34]. The MI is a brief intervention style, using a directive approach to help an individual come to a decision and find the internal motivation to modify its behavior. The MI is based on empathy, the absence of argumentation, the nonjudgmental exploration of ambivalence, and the respect of a person’s autonomy [29]. Motivation is the probability that a person will start, pursue and adhere to a specific strategy of change. The MI is based on four main principles: 1) empathy, 2) developing a discrepancy between interviewee’s current and desired behavior, 3) dealing with resistance, and 4) empowerment. The goal is to engage that person in a collaborative working relationship, allowing him to feel involved in the decision to change, in a respectful and non-judgmental atmosphere. Counseling based on MI involves five core communication strategies: 1) open-ended questions, 2) affirmative statements, 3) reflective listening, 4) careful summarization, and 5) informing and advising only when prior permission was expressly given by the interviewee to do so. Prochaska and DiClemente’s trans-theoretical stages of change model [34, 35] is a behavioral approach model. It typically features five stages over the course of behavioral change: 1) pre-contemplation, 2) contemplation, 3) preparation, 4) action and 5) maintenance (Fig. 1). In order to mobilize mothers to vaccinate, it is important to welcome them at their own respective stage regarding their current intention toward infant vaccination. The aim of the MI-based intervention is to help bring the mother to a forward stage, rather than to the final decision to vaccinate. It is fundamental to keep in mind that, for many mothers, coming to a change of opinion on vaccination is a difficult process.

The MI-based intervention covers five main areas: 1) the six diseases targeted by vaccination at 2, 4 and 6 months and their consequences; 2) vaccines and their efficacy; 3) the importance of the immunization calendar at 2, 4 and 6 months; 4) the reluctance to vaccinate and vaccination side-effects, based on the tool developed by the Centers for Disease Control and Prevention (CDC) National Immunization Program and the Frequently Asked Question section of the Quebec Immunization Protocol [36]; and 5) local vaccination services and facilities in each of the study regions.

#### Standardization

The intervention was delivered in a standard manner at all of the four participating study sites, by different research assistants at each site. A standard operating procedures manual was prepared to support research assistants’ training. This manual is a compendium of factual information about vaccines delivered to infants aged 0 to 6 months with a great attention to adapt those information in a easily and understandable way for mothers. The manual data were collected from the Quebec Immunization Protocol [36] and the Canadian Paediatric Society [37], on infant vaccines that protect against diphtheria, tetanus, poliomyelitis, whooping cough, and *Haemophilus influenzae* type b, hepatitis B, pneumococcal and rotavirus infections. Each research assistant also received the same validated training session on MI techniques. Prior to starting the study, a two-week run-in phase was conducted at each participating maternity ward in order to identify potential facilitators, obstacles and barrier(s) to implementation. During this run-in phase, the assistant coordinator and the research assistant at each participating site validated the use, under real-life conditions and situations, of the MI techniques.

For purpose of the PromoVaQ Study, we adapted the Prochaska’s trans-theoretical stages of change model taking into account the alternate positions that mothers adopt with respect to infant vaccination. Prior to the MI-based intervention, the research assistant assessed with each mother her current intention to vaccinate. “Undecided” mothers were categorized as either: a) resistant to vaccination (pre-contemplation stage) or b) open but hesitant toward vaccination (contemplation stage). “Committed” mothers were categorized as either: a) deciding to vaccinate (preparation stage) or b) mobilizing to vaccinate (action stage). The research assistant accepted mothers’ responses in a nonjudgmental manner and adjusted the MI-based intervention according to each mother’s stage and the modalities described under Fig. 1.

#### Control arm

Participants enrolled in the control arm of the study were provided with a copy of the Public health vaccine brochure handout upon hospital discharge, as per usual care.

### Study outcomes

**Fig. 2:**
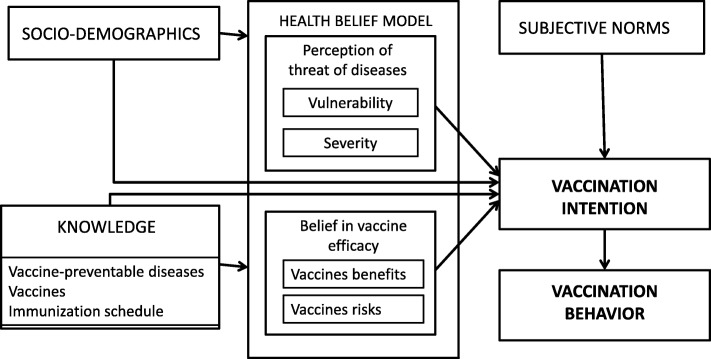
Intervention composite model

The primary outcome will be vaccine coverage at 7 months. Secondary outcomes are: 1) vaccine status (complete, incomplete or non-vaccinated) at ages 3, 5, 13, 19, 24 and 36 months; 2) median vaccination age for vaccines recommended at 2, 4, 6, 12 and 24 months; 3) mean number of days under-immunized at 2, 4, 6, 12 and 24 months; 4) maternal intention to vaccinate pre- and post-intervention; 5) maternal vaccination hesitancy score pre- and post-intervention. Independent variables are maternal socio-demographic and clinical characteristics, the perceptions and opinions toward vaccination of families having received the MI-based intervention, and components of the composite model used for the study (Fig. 2).

### Outcome measurements tools

Vaccine coverage will be calculated from data uploaded from the Eastern Townships public health vaccination registry, the provincial vaccination registry (2016 - onwards) and the postal survey of mothers of children born in Montreal-area maternity wards. To collect data on family beliefs, attitudes and intention to vaccinate, questionnaires were distributed to families pre- and post-intervention. The pre-intervention (Q1) and post-intervention (Q2) questionnaires were elaborated and built according to a composite model (Fig. 2) inspired from the Health Belief Model [38] and the Theory of Planned Behaviour [39]. Both questionnaires were self-administered and validated during the PromoVac study. Answers were provided according to a 4-category Likert scale. Q2 also collected data with respect to maternal satisfaction regarding the MI-based intervention received. Q2 was offered only to mothers having received the MI-based study intervention. Throughout the study, research assistants at each participating maternity kept a record of all implementation barriers and facilitators.

### Study schedule and follow-up scheme

In order to give a reasonable time frame for mothers to have their infants vaccinated as per Quebec’s recommendations at 2, 4 and 6 months of life, the vaccination status of each participating infant was assessed at ages 3, 5 and 7 months. This one-month period is set by the Canadian Network of Immunization registries [8]. Vaccine status is considered “complete” when an infant has received all vaccines and antigens recommended by the Quebec Immunization Protocol. In order to benefit from the most exhaustive data possible, vaccination data of all participating children will be uploaded from the registries at two separate time points i.e. at 10 and 40 months passed the inclusion of the last infant in the study, enabling collection of the 2, 4 and 6 months vaccine data, and next the 13, 19, 24 and 36 months vaccine data. Once the vaccine status of each child will be determined, percentage of vaccine coverages will be calculated for each study population (intervention/control) according to the following formula: the number of infants in the study population (intervention/control) displaying “complete” vaccine status (at 3, 5, 7, 13, 19, 24 and 36 months) divided by the total number of infants in the study population (intervention/control) during the study period, multiplied by 100.

### Sample size

The 2012 survey estimated vaccine coverage was 74% at 7 months for Québec infants [40]. Our hypothesis is that the intervention will improve vaccine coverage by a minimum of 5% raising it to 79% for mothers having received the MI-based intervention at the maternity ward. A total of 1128 participants are needed to detect a significant difference of 5% in vaccine coverage at 7 months of age, with a risk of error α = 0.05, a power of 80% and using a bilateral test. Factoring a 20% rate of attrition, notably for the Montreal-area for which there is no vaccination registry, a total of 2750 mothers were included in this study i.e. 625 from the Montreal-area and Québec city sites, and 875 from the Sherbrooke site, in order to compensate for follow-up losses at both Montreal-area sites.

### Analytical statistical plan

Files containing vaccination data on participants and the questionnaires from each site will be sent to the Institut National de Santé Publique du Québec (INSPQ) for centralized data capture and analyses. Descriptive analyses of answers provided from each of the two study questionnaires will be performed. Primary analyses will be performed under intention to treat, whereas sensitivity analyses will include per protocol analyses. Frequencies and percentages will be presented as categorical variables. Means and standard deviations will be presented for normally-distributed continuous variables. Medians and interquartile ranges will be presented for abnormally-distributed continuous variables. Answers to the pre- and post-intervention questionnaires, notably the trans-theoretical stages of change model components, as well as the intention to vaccinate measured pre- and post-intervention will be compared using the McNemar’s test for categorical variable and Wilcoxon signed-rank test for continuous variable. Pre-intervention intention to vaccinate will then be dichotomized (“Certainly” answers, defined as certain, vs. “Probably”, “Probably not” and “Certainly not” answers, defined as uncertain) and compared by univariate analysis to the other variables as measured pre- and post-intervention. Categorical variables and vaccination intention will be compared using the Khi2 or Fisher exact test (expected frequency < 5). Normally-distributed continuous variables will be compared to intention to vaccinate using Student’s T-test and abnormally-distributed continuous variables will be compared to intention using the Mann-Whitney test. To identify the principal influencing factors on the “certain” intention to vaccinate, a multivariate logistic regression model with a step-by-step method will be used. To avoid co-linearity between the model’s independent variables, only those variables with a univariate *p* < 0,1 and a correlation coefficient < 0,6 (Pearson correlation, Spearman correlation) will be integrated into the multivariate logistic regression model. Co-linearity will be checked for the final model, and model fit will be assessed using the Akaike Information Criterion and the Hosmer and Lemeshow test. The vaccination status of the children will be compared between the intervention and the control groups. Missing data will be handled through multiple imputation techniques. All statistical will be 2-tailed and *p*-values of 0.05 or less will be considered significant.

### Trial oversight

Dr. Arnaud Gagneur MD and his research team are in charge of the study oversight. Quality control of the MI-based study intervention was audited across each of the four study sites by the same research coordinator, both at the beginning and during the recruitment period. Recruitment progress at each site was monitored in real time using the study randomization system. Double capture of study questionnaire answers, data management and analyses will be centrally performed by the INSPQ.

### Risk of bias mitigation strategies and security assessment

In the PromoVaQ trial, the Hawthorne effect will be limited by the fact that each group will receive either the MI-based study intervention or the standard-care handout. However, previous studies on handouts as a means to promote vaccination have shown this strategy to be inefficient [23]. The study group receiving the handout will thus be considered the control group. The risk of contamination is low as the intervention will be dispensed by the research team as opposed to the healthcare team. Research assistants may bear a favorable bias toward the intervention group; however this risk is considerably mitigated by the fact that the control group will receive the handout on childhood vaccination without any further explanation. Therefore, the risk of actual bias in favor of the study intervention is rather weak. Since the study is unblinded to the research team, the medical team and the participants, no other co-intervention that may impact the intervention will be delivered. An additional strategy to ensure data quality is that the primary outcome will be validated by third parties, namely the Eastern Townships public health authorities, the provincial vaccination registry and the postal survey on mothers of children born in Montreal-area maternity wards. These parties and the INSPQ biostatistician will all be blinded to the study. In order to thoroughly document the inclusion of participants, a family registry will be set up. Reasons volunteered by families who decline to participate in the study will also be documented in this family registry. Based on the pragmatic and secure nature of the study, an independent Data Safety Monitoring Board (DSMB) was deliberately excluded. Therefore, the absence of interim analyses will help avoid multiplicity of analysis.

## Discussion

As mentioned above, prior to their study participation and randomization, written informed consent will be obtained from each participant, including their authorization to communicate with public health authorities in order to upload data concerning their child from vaccination registries (for participants from the Eastern Townships and the Quebec-City region) or to receive survey data (for those from the Montreal-area). The study received approval from the Institutional Research Ethics Review Boards of all four sites, and respective participating maternities agreed to participate in writing the protocol. This study is registered online (as January 2016) at www.clinicaltrials.gov (NCT02666872). Clinical equipoise exists for this pragmatic RCT as the expert scientific community is genuinely uncertain as to which strategy is superior to the other in promoting infant vaccination.

The PromoVac regional demonstrated the feasibility and efficacy of the concept of a MI-based vaccination promotion intervention delivered to mothers at the maternity ward on the day following delivery and birth. Scaling up of the study and validation of its concept at the provincial level is necessary to determine the relevance of integrating MI-based strategies into the Quebec Vaccination Promotion Program. Ultimately, such a strategy could provide the provincial health and social services ministry (MSSS) with efficient solutions to improve provincial vaccine coverage. Should results of the regional study be confirmed by this RCT, serious consideration should then be given by Ministry of Health authorities to the possible implementation of MI-based strategies across provincial maternity wards. Our team includes clinicians, academic researchers, community health physicians, members of the Quebec Immunization Committee (CIQ), and members of the Institut National de Santé Publique du Québec (INSPQ) Vaccination Promotion Committee. We are therefore in a privileged position to ensure the rapid dissemination of our research results to experts of the scientific community as well as their immediate transfer to decision-makers. Key scientific messages will be disseminated through presentations at national and international venues, as well as through scientific publications in peer-reviewed journals. Pertinent results for health authorities will be made available in real time to the Ministry of Health through our network of liaisons and to each member to the project via the CIQ and the INSPQ Vaccination Promotion Committee. This study’s results will also be formally presented to regional public health authorities and, at the provincial level, to the CIQ and to the INSPQ Vaccination Promotion Committee. Should the MI-based strategy one day prove successful as part of the Quebec Vaccination Promotion Program, the next logical step would be for the National Advisory Committee on Immunization (NACI) to consider MI-based intervention implementation to the rest of Canada. As member-president of the NACI and co-investigator on this RCT, Prof. C. Quach will relay the study results to this Committee. Ultimately, this MI-based vaccination promotion strategy could contribute to curbing the morbidity and mortality rates of vaccine-preventable diseases, as well as their associated health costs and burden for human society. Once proof-of-principle has been established in the Province of Quebec, our MI-based vaccination promotion strategy is expected to sustain wide international interest.
